# Evaluating website resources shared online amongst women with intimate partner violence experiences: analysis of an online health community

**DOI:** 10.3389/fpsyg.2026.1750050

**Published:** 2026-02-16

**Authors:** Vivian Hui, Lidan Tian, Malavika Eby, Bohan Zhang, Rose E. Constantino

**Affiliations:** 1 Center for Smart Health, School of Nursing, The Hong Kong Polytechnic University, Hong Kong, Hong Kong SAR, China; 2 Health and Community Systems, School of Nursing, University of Pittsburgh, Pittsburgh, PA, United States; 3 Sidney Kimmel Medical College, Thomas Jefferson University, Philadelphia, PA, United States

**Keywords:** help-seeking behaviors, intimate partner violence, medical informatics, online health communities, violence against women, website-sharing

## Abstract

**Introduction:**

Intimate partner violence (IPV) affects around one in four women globally, posing substantial health risks. IPV survivors often consult online health communities for anonymous assistance rather than formal services. Though online health community members frequently share websites to answer questions, no studies have investigated the characteristics and relevance of websites shared in IPV online health communities. This study aims to identify the categories of websites shared in IPV online health communities and evaluate associations between post characteristics and website relevance to survivors’ help-seeking needs.

**Methods:**

Data were extracted from posts and comments on the r/domesticviolence, a subreddit (topic-specific community) dedicated to domestic violence support on Reddit (a social media platform), from November 2020 to November 2021. We included English-language posts seeking advice, written by adult women with IPV experiences, with at least one website shared in the comments. Website links were annotated by topics and categorized as relevant or irrelevant to help requested by original posters. Posts were annotated for characteristics including post length, mentions of “red flags” for lethality, and specific versus general help requests. Chi-square and *t*-test were used to determine association between post characteristics and websites’ relevance.

**Results:**

A total of 170 website links were categorized into eight themes, with General IPV Resources and Support (32.4%) and Understanding IPV (28.2%) being the most common. Approximately 75.3% of the websites were relevant to the types of help sought by original posters. Post characteristics showed no significant association with the relevance of the websites.

**Conclusion:**

This study sheds light on the types of websites shared within IPV online health communities and informs IPV agencies and clinicians about the addressed and unaddressed needs of women IPV survivors seeking help online. These findings could help optimize the design of online health community platforms, including digital tools that automatically suggest relevant websites to IPV survivors.

## 1 Introduction

Intimate partner violence (IPV), which comprises physical, emotional, financial, or sexual violence between intimate partners, is a serious human rights and public health issue that affects over 736 million women worldwide (Kyle, 2023; World Bank Gender Data Portal, n.d.). Globally, approximately one in four women have experienced physical or sexual IPV in their lifetime (Sardinha et al., 2022), and IPV is the leading cause of nonfatal injuries in women (Lutgendorf, 2019). The global lifetime prevalence of IPV in women ranges from 15 to 71% (Stewart and Vigod, 2019). IPV poses serious physical consequences for women, such as physical injury and disability, sexual trauma, and homicide, as well as emotional consequences, including disordered attachment styles, depression, anxiety, post-traumatic stress disorder, and suicidal ideation (Lysova et al., 2019; Scheffer Lindgren and Renck, 2008; Ellsberg et al., 2008). Women with IPV experiences may also be subjected to financial problems and isolation from friends and family members (Lin et al., 2023). These consequences hinder survivors and victims from effectively identifying IPV, seeking help, and escaping their abusive partners (Heron and Eisma, 2021).

Seeking appropriate formal and informal support significantly enhances recovery and safety outcomes of IPV survivors, with research demonstrating reduced psychological burden, decreased trauma symptoms, and improved wellbeing following effective intervention (Wright et al., 2022; Liang et al., 2005; Ullman and Peter-Hagene, 2014). However, IPV is still grossly underreported, with only 25% of IPV survivors reporting their experiences to the police, and only 21% receiving medical care after an IPV incident (Whitton et al., 2023). Fear of judgement and stigma hinder survivors’ ability to disclose abuse and seek formal help (Liang et al., 2005; DeKeseredy and Schwartz, 2008). Recent cross-cultural research has demonstrated that cultural stigma leads survivors to perceive their abuse as a private matter, diminishing their willingness to seek external support and consequently exacerbating adverse mental health outcomes (Li et al., 2025). During the rise of the #MeToo movement, many women who publicly disclosed IPV faced criticism for not reporting their abuse to institutional authorities such as police or courts sooner (Ullman and Peter-Hagene, 2014; Gurm et al., 2020). In fact, many survivors decided to formally file complaints after significant delays, waiting weeks, months, or even years, while others chose not to file at all. Prior studies indicate that formally disclosing IPV presents substantial barriers, as survivors frequently encounter re-victimization, blame, or neglect by biased authorities, in addition to lengthy and agonizing court battles (Gezinski and Gonzalez-Pons, 2022). Furthermore, woman IPV survivors with disabilities often receive inadequate care from healthcare providers and lack access to resources tailored to their needs (Alhusen et al., 2020). Survivors who distrust formal systems or experience self-blame increasingly turn to anonymous online platforms as alternative spaces for disclosing their experiences (Stewart et al., 2024). Specifically, they frequently share their IPV experiences with peer networks where they are accepted and empowered with compassion and non-judgment (Parvin et al., 2016).

With the growth of the Internet, thousands of IPV survivors currently turn to online health communities, where social support and health information resources are accessible in an anonymous manner (Perry et al., 2020). Online health communities are online groups focused on specific health concerns that exist on major social networking platforms, such as Facebook, Twitter/X, and Reddit. In recent years, Online health communities have become popular spaces for women with IPV experiences to disclose their stories and seek resources and advice from other survivors, especially during the pandemic, since people spent more time online (Tanis, 2008). Lyons and Brewer (2022) reported that survivors share personal anecdotes of IPV in online health communities, including factors that heightened their distress and preparedness to leave. Hui et al. (2023) analyzed the types of help offered in online health communities to women with IPV experiences and found that 71.2% of help-seeking posts received responses from online health community users sharing their experiences, mistakes, and lessons learned. IPV online health communities enable survivors to exchange information tailored to their needs, post and answer questions, and learn from others’ experiences without actively posting or commenting (Tanis, 2008; Carron-Arthur et al., 2015). The resources shared among users can benefit both IPV survivors and professionals by providing knowledge about key aspects of IPV, including how to identify abuse, address trauma, and promote recovery. Among the shared resources, website links sharing is a primary information in online health communities that directs users to external resources, such as crisis hotlines, shelters, and legal aids (Thaker et al., 2022; Chuang and Yang, 2014). It connects users to actionable resources beyond peer advice. The quality of shared websites plays a crucial role in guiding survivors toward effective help-seeking experience.

While several studies have examined how information or experiences are shared in online health communities, few studies have focused on the association between the information requested by posters and information provided by online health community users. Bizzotto et al. (2023) revealed that 26.1% of comments in mental health online health communities contained medically inaccurate information and almost 60% of posts presented at least one piece of incorrect information. Thaker et al. (2022) conducted a study of ovarian cancer online health communities which discovered that only about 48.3% of shared resources could be identified as relevant to original posters’ questions. Therefore, IPV survivors who depend on resources shared in online health communities to protect their emotional and physical safety could be inadvertently exposed to misinformation, victim-blaming, or other irrelevant, biased, or harmful information (Grimani et al., 2020). Analyzing whether the websites shared in IPV OHCs are relevant to the questions posed by OPs will show which resources can meet the information needs of IPV survivors, empower OPs to find improved success in receiving relevant resources, and validate the effectiveness of OHCs in fulfilling the help-seeking needs of IPV survivors.

Prior research has investigated the circulation of website resources amongst online health community members in some health domains, such as ovarian cancer, heart disease, and diabetes, but not IPV (Thaker et al., 2022; Nath et al., 2016). Therefore, the goal of our study is to shed light on the topics and usefulness of website links shared in IPV online health communities. Particularly, this study aims to identify 1) the categories and domains of websites shared in IPV online health communities, 2) the relevance of websites to the types of help sought by original posters, and 3) whether website relevance is associated with any post characteristics (e.g., post length). These results can be utilized by clinicians, researchers, and IPV agencies to gauge the landscape of peer education in IPV online health communities and determine to what extent online health community resources are reliable or factually accurate enough to refer to IPV survivors.

## 2 Materials and method

### 2.1 Study design

This is an exploratory descriptive study to characterize websites shared in IPV online health communities and their relevance to original posters’ help-seeking needs.

### 2.2 Data collection

Data were collected from November 14, 2020, to November 14, 2021, from Reddit, a social networking site. Reddit contains an array of subreddit communities where users post questions and announcements about specific topics, including r/domesticviolence, our subreddit of focus (Reddit, n.d.a). We used the Python 3.8 programme package to extract post and comment data from r/domesticviolence, after which we crawled the URLs of all websites for in-depth analysis. Data was collected using PRAW (Python Reddit API Wrapper), which provides programmatic access to Reddit’s API. We used packages like pandas, seaborn for data processing.

The questions posed in “r/domesticviolence” cover a number of IPV topics, such as determining whether a person’s relationship is abusive or healthy, or gathering advice on how to develop a safe escape plan. These online exchanges among IPV survivors likely inform and influence their decision-making and self-understanding. As of July 2025, there are more than 46,000 members present in r/domesticviolence. Online health community users provide support to help-seeking original posters by commenting personal anecdotes, emotionally supportive words, hotlines, video links, and website links (Hui et al., 2023). These shared websites typically include IPV-focused organizational resources (e.g., national hotlines, shelter directories), educational materials about abusive dynamics, and legal or safety planning information.

Reddit was selected as the data source with the following rationales: 1) Reddit allows users to express their needs without word and character limits. 2) Users have greater anonymity on Reddit than on other social media websites. They are not required to provide their names, locations, or other identifying information on their Reddit profiles, allowing them to share sensitive information more freely in posts and comments.

### 2.3 Inclusion and exclusion criteria

A total of 1,996 postings were identified that met these inclusion criteria: 1) self-disclosure as a woman over 18 years of age, 2) description of personal experiences with IPV, and 3) an explicit request for help, as evidenced by at least one question. All advertisements or announcement posts, posts written by non-abused and/or underaged and/or male users, and non-English-language posts were excluded from the sample. While the help-seeking posts were categorized and analyzed for another research paper, this study extracted websites from the posts’ comment sections for further analysis (Hui et al., 2023). All posts in the final sample contained at least one website link shared by an online health community member in its comment sections. “Resource relevance” refers to whether content shared by users in comment sections appropriately addresses the information requested by a poster. If a resource fails to address the type of information sought by the original poster, we deemed it irrelevant (Cosijn and Ingwersen, 2000).

Two websites were inaccessible during the annotation process, likely due to web content getting taken offline or undergoing construction. Thus, they were excluded from our resource type distribution analysis.

### 2.4 Data structuring, codebook development, and annotations

We extracted the original poster’s username, user number, post URL, post title, post content, and number of comments from each post in our sample using the Python 3.8 Programme package. To ensure participant confidentiality, all usernames were replaced with unique, randomly generated user IDs throughout the analysis.

### 2.5 Website annotations

Two coders manually reviewed all posts and compiled a list of websites from each comment section. These websites were then examined and annotated for their main content topics by the same coders. The final list of topics was narrowed to semi-specific categories, including “Legal/Government-based Support,” and “Understanding IPV.” These categories were used to classify both the types of help requested by original posters and the content of shared websites, enabling us to assess the relevance of shared resources to survivors’ expressed needs. These codes were then employed to annotate a larger sample of websites. Websites that did not fit existing categories led to the creation of new codes.

After revision, the final thematic analysis consisted of eight categories (Table 1), which were used to annotate all websites by topic. Subsequently, both coders classified all sample websites according to website type using a codebook adapted from an established classification framework for health-related websites (Thaker et al., 2022) (Table 2).

**Table 1:** Categories of website content in r/domesticviolence.

Website Category	Websites, *n* (%)	Definition	Most shared website URL
General IPV Resources and Support	55 (32.4)	Lays out overviews of IPV agencies and nonprofits, hotlines, live chat platforms, and virtual support groups.	https://www.thehotline.org
Safety Warnings	9 (5.3)	Provides information about risk factors for homicide, most often choking or strangling. Frequently references “warning signs” for impending dangerous, potentially lethal events.	https://www.strangulationtraininginstitute.com/strangulation-the-red-flag-of-domestic-violence-that-we-never-discuss/
Financial, Healthcare, Housing + Food Support	14 (8.2)	Offers logistical information for survivors about how and where to access financial aid, healthcare, housing, and food.	https://www.domesticshelters.org
Escaping an IPV Situation	11 (6.5)	Presents information on different aspects of how to plan and complete a safe escape from an abusive relationship.	https://www.ncdsv.org/images/dv_safety_plan.pdf*
Understanding IPV	48 (28.2)	Explains IPV- and abuse-related topics like trauma bonding, “reactive abuse,” BPD, stages of abuse, and perpetrators’ motives.	Online copies of the book, Why Does He Do That, by Lundy Bancroft
Legal/Government-based Support	14 (8.2)	Offers legal guidelines and recommendations relevant to IPV survivors, often focusing on custody of kids and legal protections for survivors of abuse.	https://www.rosen.com/domestic/co-parenting-with-abuser/
Emotional Support	10 (5.9)	Consists of encouraging and warm messages to help IPV survivors stay resilient, find courage, feel emotionally affirmed, hold onto hope, and recover from emotional wounds.	https://www.hope4-recovery.org/program.html ^*^
Miscellaneous	9 (5.3)	“Niche” websites that provide information in highly specific and unique domains outside the seven other thematic categories (e.g., pet shelters, apps, and safety technology).	https://www.safehavensforpets.org

^*^These links were not accessible at the time of manuscript preparation.

**Table 2:** Codebook used to identify types of online health resources.

Code	Description	Sample website URLs
IPV informational resources	Webpages containing focused information on a specific IPV-related topic aimed toward IPV survivors, such as health articles, health expert blogs, and health topic information websites.	https://www.strangulationtraininginstitute.com/strangulation-the-red-flag-of-domestic-violence-that-we-never-discuss/ https://www.healthline.com/health/mental-health/trauma-bonding
IPV news reports	Webpages presenting IPV-related news, such as articles about IPV incidents, or news updates of IPV-related laws or policies.	https://www.wreg.com/news/study-domestic-abuse-victims-10-times-more-likely-to-be-killed-if-suspects-choked-them-in-past/ https://www.spotlightonwomenandviolence.com/2017/04/29/choking-seen-as-prelude-to-murder/amp/
IPV survivor educational resources	Resources provided by governments and IPV organizations, including IPV survivor guidelines, factsheets, and pamphlets.	https://www.dangerassessment.org/uploads/DA_NewScoring_2019.pdf https://www.msktc.org/tbi/factsheets/understanding-and-coping-irritability-anger-and-aggression-after-tbi
Academic literature	Research articles and studies relevant to IPV and its effects.	https://www.ncbi.nlm.nih.gov/pmc/articles/PMC4814782/
Web-based social groups	Webpages containing user discussions and posts on web-based communities, question-answering forums, and social networking sites.	https://www.reddit.com/r/abusiverelationships/ https://www.old.reddit.com/r/domesticviolence/comments/jtzpg3/does_anyone_have_any_experience_with_domestic/
IPV organizations	Webpages referring to the home page of an IPV organization, nonprofit institute, or government website.	https://www.1in6.org/ https://www.victimconnect.org https://www.thehotline.org/
IPV survivor blogs	IPV survivor- or caregiver-generated personal websites and blogs.	None
E-Commerce	Webpages that are online shopping sites or dedicated to product promotions or advertisements.	https://www.hollieguard.com/
Videos	Links to video-based content.	https://www.youtube.com/watch?v=V1yW5IsnSjo https://www.youtu.be/6zCJ-olN3lI
Non-IPV articles	Webpages dedicated to content outside of the IPV domain.	https://www.freefinancialhelp.net/where-to-find-help-with-bills/ https://www.irs.gov/identity-theft-fraud-scams/get-an-identity-protection-pin

### 2.6 Post annotations

During the initial manual review, 3 post characteristics were identified: (1) Mentions of “red flags” for IPV lethality, like choking or strangling. Choking and strangling were specifically included because they are both critical risk factors for potential escalation of violence and even homicide (Campbell et al., 2003). (2) Specific versus general requests, where specific requests were coded when the original poster clearly outlined the types of help they were seeking (e.g., asking for legal resources, housing options, or information on a particular aspect of IPV). General requests were coded when the original poster described their help-seeking needs but did not specify the exact type of support. (3) Mentions of original poster wanting to return to their abusive partner. Our final codebook consisted of nine variables (Table 3). All posts in our sample were manually annotated using this codebook by two coders, and the interrater agreement was calculated by Cohen’s Kappa (*κ*).

**Table 3:** Codebook used to classify characteristics of help-seeking posts by IPV survivors.

Variable	Codes	Definition
Post ID		Randomly generated alphanumeric ID assigned to all sample posts.
Post length	1, 2	Post word length is either above (1) or below (2) the average length of all posts in our sample (298 words).
Mention of “red flag” for IPV lethality such as choking or strangling	1, 2	Original poster mentions (1) or does not mention (2) that she was choked or strangled by an abusive partner in her post.
Specific or general question	1, 2	Original poster either clearly outlines what type of help she is seeking from online health community members by asking a specific question (1), or generally states that she is seeking any form of help/support after describing her situation (2).
Mention of original poster wanting to return to their abusive partner	1, 2	Original poster mentions (1) or does not mention (2) at least one sentence expressing a desire or plan to return to her abusive partner in her post.
Website URL (from comments)		URL of the website.
Number of websites shared in comments	#	The number of websites shared by online health community users (not including original poster) in a post’s comment section.
Type(s) of help requested by original poster	1–8	Help-seeking post was coded for one or several of the following categories: (1) General IPV Resources and Support; (2) Understanding IPV; (3) Financial, Healthcare, Housing, and Food Support; (4) Safety Warnings; (5) Legal/Government-based Support; (6) Escaping an IPV Situation; (7) Emotional Support; and (8) Miscellaneous.
Type(s) of help provided by website	1–8	Website content was coded for one or several of the following categories: (1) General IPV Resources and Support; (2) Understanding IPV; (3) Financial, Healthcare, Housing, and Food Support; (4) Safety Warnings; (5) Legal/Government-based Support; (6) Escaping an IPV Situation; (7) Emotional Support; and (8) Miscellaneous.
Relevance of the website to original poster’s needs	1, 2	The topic of the website does (1) or does not (2) match at least one of the topics of help requested by original poster in her post.

### 2.7 Ethics approval

This study obtained approval for exemption from the Institutional Review Board at the University of Pittsburgh (MOD20030179-002) as the data on Reddit is publicly available. The research team noticed that the r/domesticviolence subreddit adopted new policies prohibiting research data scraping following a change in its moderation team. At the time of our IRB approval in November 2020, the subreddit did not have published rules prohibiting research. The current moderation team has confirmed with the corresponding author that the policy change occurred after their transition.

Our study complied with Reddit’s Terms of Service and subreddit policies in effect at the time and was approved by our institution’s IRB prior to data collection. We analyzed only publicly available posts, engaged in no user interactions, and de-identified the data. Our university IRB reconfirmed in 2025 that the study involved no misconduct. As the data policy could evolve, future researchers may need to reconfirm the data availability with subreddit moderators.

### 2.8 Statistical analysis

A chi-square test was used to assess the associations between categorical post characteristics—such as mentions of choking/strangling, specific versus general requests, and mentions of original poster wanting to return to their abusive partner—and the relevance of websites shared by commenters. The chi-square test is appropriate for examining relationships between categorical variables (McHugh, 2013). For example, we examined whether posts that mentioned risk factors for homicide, like choking, would prompt commenters to share websites that discuss risk factors for lethality in IPV situations or resources to promote original poster’s safety. A *t*-test and Mann–Whitney *U* test were used to evaluate the same question with quantitative post characteristics, like post length by word count. The *t*-test is appropriate to compare means between two groups, while the Mann–Whitney *U* test was employed as a non-parametric alternative when data did not meet normality assumptions (Kim, 2015). SPSS (version 28) was used to perform statistical analysis, with a significance level of 0.05. *Post-hoc* power analyses were conducted using G*Power 3.1 to estimate the statistical power of our analyses given the sample size.

## 3 Results

A total of 1,996 postings were collected from Reddit. Prior to screening, posts with incomplete content were excluded. During initial screening, postings were assessed against inclusion criteria (self-identified women over 18, description of IPV experiences, and explicit help-seeking request). Postings meeting these criteria were further screened to identify those containing at least one website link shared by commenters. After this process, 78 postings with 170 website URLs in total were selected for subsequent in-depth analysis.

### 3.1 Website topics and domains

**Figure 1:**
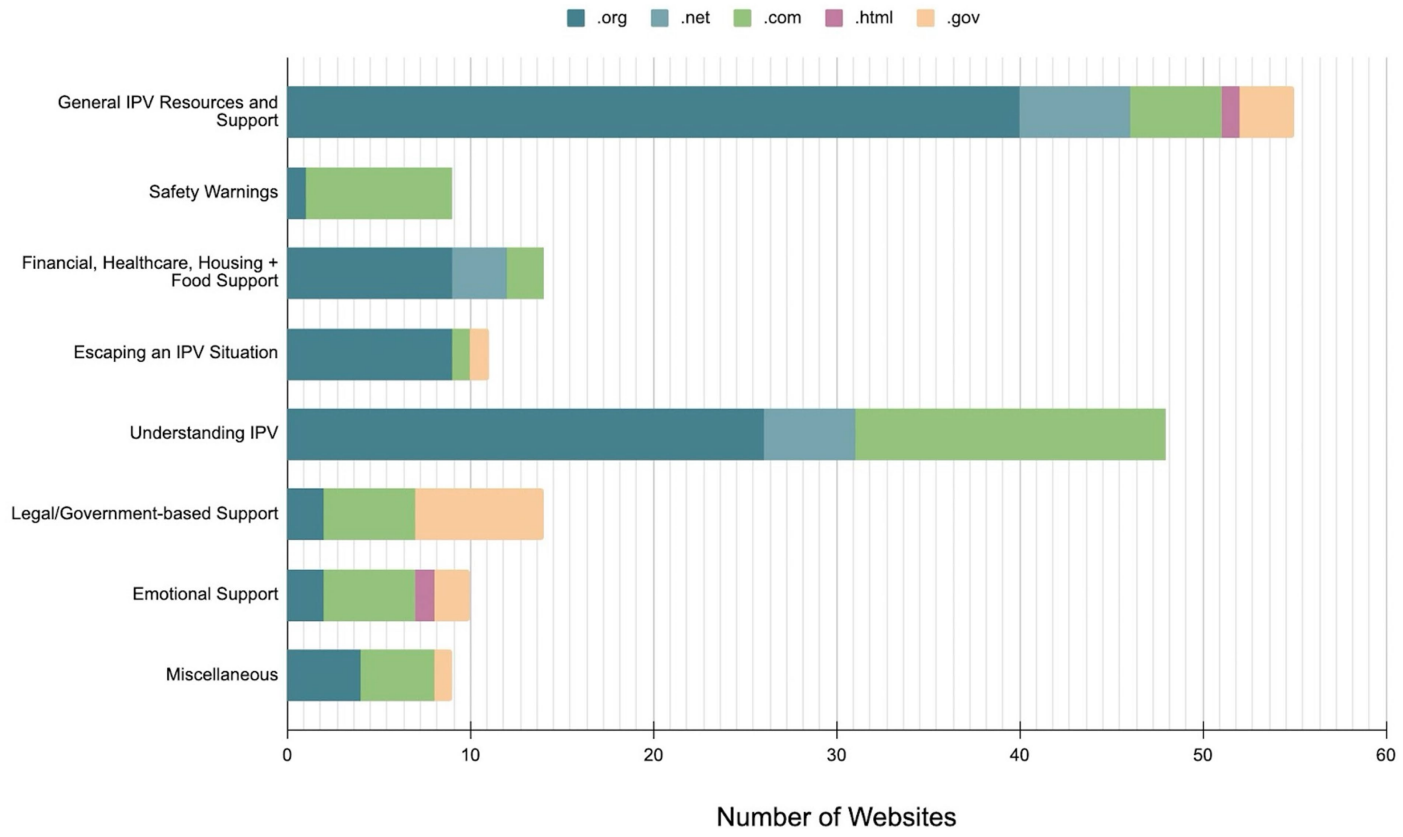
Website domains represented across eight categories of website-sharing in an IPV online health community.

Websites shared by online health community commenters were categorized into eight types of assistance. The two most prevalent categories were “General IPV Resources and Support” (*n* = 55, 32.4%) and “Understanding IPV” (*n* = 48, 28.2%), together accounting for over 60% of all shared resources. The remaining six categories included: Safety Warnings (*n* = 9, 5.3%); Financial, Healthcare, Housing, and Food Support (*n* = 14, 8.2%); Advice to Escape an IPV Situation (*n* = 11, 6.5%); Legal/Government-based Support (*n* = 14, 8.2%); Emotional Support (*n* = 10, 5.9%); and Miscellaneous (*n* = 9, 5.3%) (Table 1). All websites held a .org, (non-profit organizations) .com, (commercial websites) .gov, (government websites) or .net (network services) domain, with their distribution within each category reflected in Figure 1. The most frequently shared website was https://www.thehotline.org, the National Domestic Violence Hotline, which provides information on IPV agencies, nonprofits, hotlines, and safety planning resources. Other commonly shared resources included https://www.domesticshelters.org for housing support, and Lundy Bancroft’s book *Why Does He Do That* for understanding abusive dynamics. The majority of shared websites were US-based resources.

The most common top-level domain (TLD) for URLs was .org, representing 54.7% (93/170) of all shared resources, followed by .com, .gov, .net, and .html. Table 4 details the number of website links shared in each TLD and the percentage of links that are relevant or irrelevant to original posters’ needs. The association between TLD and the relevance of websites was analyzed using a chi-square test and the results showed that there was no association between TLD and the relevance of websites to original posters’ needs (*χ*^2^ = 5.788, *p* = 0.199).

**Table 4:** Top-level domain (TLD)-based distribution of shared websites, and percentage of websites that are relevant to original poster’s needs (*N* = 170 links).

Top-level domain (TLD)	Links, *n* (%)	Relevant to original poster’s needs (*n* = 128), *n* (%)	Irrelevant to original poster’s needs (*n* = 42), *n* (%)	*χ* ^2^	*p*
.com	47 (27.6)	40 (31.3)	7 (16.7)	5.788	0.199
.gov	14 (8.2)	11 (8.6)	3 (7.1)		
.html	2 (1.2)	2 (1.6)	0 (0.0)		
.net	13 (7.6)	8 (6.3)	6 (14.3)		
.org	93 (54.7)	67 (52.3)	26 (61.9)		

### 3.2 Website-sharing outcome

Approximately 75.3% (*n* = 128) of websites shared by online health community members were relevant to the type of help originally sought by original posters in their respective posts. This outcome was not found to be promoted significantly by any of the post characteristics assessed.

### 3.3 Resource type categorization

To move beyond simple domain-based analysis, we manually checked each link and categorized shared resources into content-focused categories. Two coders (ME and BZ) coded the links independently, creating the coding scheme in Table 2. The inter-annotator agreement between the two coders was Cohen Kappa (*κ*) = 0.702, with a substantial agreement of 75.88%.

Table 5 shows the distribution of websites based on resource types drawn from Thaker et al. (2022). As two links in our sample later presented as unavailable, we included all 170 links for our domain analysis, but our content analysis was limited to 168 links. Webpages by IPV-related organizations, usually homepages, were the most popular type of shared resources, accounting for 31.4% of the shared links. Next were IPV-related articles with 24.4% of shared links, and third were educational resources for IPV survivors (23.2%). The correlation between resource type and website relevance to original poster’s needs was analyzed using the chi-square test. It was found that there was no correlation between resource type and website relevance.

**Table 5:** Resource type distribution of shared websites, and percentage of websites that are relevant to original poster’s needs (*N* = 168 links).

Resource type	Links, *n* (%)	Relevant to original poster’s needs (*n* = 127), *n* (%)	Irrelevant to original poster’s needs (*n* = 41), *n* (%)	*χ* ^2^	*p*
IPV informational resources	41 (24.4)	33 (26.0)	8 (19.5)	5.073	0.773
IPV news reports	3 (1.8)	3 (2.4)	0 (0.0)		
IPV survivor educational resources	39 (23.2)	28 (22.0)	11 (26.8)		
Academic literature	1 (0.6)	1 (0.8)	0 (0.0)		
Web-based social groups	10 (5.8)	8 (6.3)	2 (4.9)		
IPV organizations	54 (31.4)	40 (31.5)	14 (34.1)		
IPV survivor blogs	0 (0.0)	0 (0.0)	0 (0.0)		
E-Commerce	1 (0.6)	1 (0.8)	0 (0.0)		
Videos	3 (1.7)	1 (0.8)	2 (4.9)		
Non-IPV articles	16 (9.3)	12 (9.4)	4 (9.8)		

Table 6 presents the characteristics of help-seeking posts and the proportion of posts with each characteristic that received relevant website resources. Statistical analysis using chi-square tests and t-tests revealed no significant associations between post characteristics and website relevance (all *p* > 0.05). *Post-hoc* power analysis indicated that with 78 posts, *α* = 0.05, and df = 1, our study had 75.5% power to detect a medium effect size (*w* = 0.3), but only 43% power to detect a small-to-medium effect (*w* = 0.2). The non-significant findings may therefore reflect either the absence of true associations or the presence of small effects that our sample was underpowered to detect. Descriptive statistics are reported below for exploratory purposes and should be interpreted with caution given the lack of statistical significance and small sample sizes within subgroups. Among posts mentioning the original poster’s desire to return to their abusive partner, 90% (9/10) received relevant resources. Only 73.3% (11/14) of posts containing red flags for lethality risk (i.e., choking or strangling) received relevant websites. Posts describing sexual abuse and financial abuse were met with lower rates of receiving relevant resources (61.5 and 57.1%, respectively) compared to posts mentioning physical abuse (80.0%) and emotional abuse (77.5%). Posts with clear, specific questions received relevant websites in 75.0% (42/56) of cases, while 95.5% (21/22) of posts with general requests received relevant resources.

**Table 6:** Characteristics of post content, and percentage of posts that receive relevant websites (*N* = 78 posts).

Post characteristic	Code	Posts receiving relevant websites (*n* = 63), *n* (%)	Post receiving irrelevant websites (*n* = 15), *n* (%)	*χ*^2^/*t*	*p*
Post length	Above the average length (298 words)	30 (47.6)	4 (26.7)	2.163	0.141
	Below the average length	33 (52.4)	11 (73.3)		
Mention of “red flag” for IPV lethality such as choking or strangling	Yes	11 (17.5)	3 (20.0)	0.000	1.000
	No	52 (82.5)	12 (80.0)		
Specific vs. general request	Specific	42 (66.7)	14 (93.3)	3.040	0.081
	General	21 (17.8)	1 (4.2)		
Original poster trying to go back to perpetrator	Yes	9 (14.5)	1 (6.7)	0.147	0.701
	No	53 (85.5)	14 (93.3)		
Type of abuse	Physical abuse	36 (80.0)	9 (20.0)	0.041	0.840
	Sexual abuse	8 (61.5)	5 (38.5)	2.377	0.123
	Emotional abuse	31 (77.5)	9 (22.5)	0.565	0.452
	Financial abuse	4 (57.1)	3 (42.9)	1.345	0.246
Number of websites shared in comments (mean ± SD)		2.25 ± 2.02	2.13 ± 1.69	0.214	0.831

## 4 Discussion

### 4.1 Principal findings

This study examined the categories and domains of shared websites in IPV online health communities, and explored the relevance between shared websites and original posters’ needs. Three key findings emerged from our analysis. First, 75.3% of shared website resources were relevant to original posters’ expressed needs, demonstrating that online health community members largely provide appropriate information that addresses IPV survivors’ help-seeking requests. Second, the distribution of website topics revealed a clear prioritization pattern, with “General IPV Resources and Support” (32.4%) and “Understanding IPV” (28.2%) dominating the shared resources, while critical safety planning resources constituted only 11.8% of shared links. Third, our analysis found no statistically significant associations among post characteristics, website characteristics, and the relevance of resources to original posters’ needs.

### 4.2 Categories of shared website resources

The distribution of website topics revealed two major categories: “General IPV Resources and Support” (32.4%) and “Understanding IPV” (28.2%). This pattern indicates online health community members prioritize comprehensive resource hubs and educational content about abuse dynamics when responding to help-seekers. However, formal resources represent only one dimension of the support provided in these communities. Beyond sharing external websites, online health community members also draw extensively on their personal experiences to help others. Previous research showed that 71.2% of help-seeking posts in IPV online health communities receive responses containing users’ personal experiences and lessons learned (Hui et al., 2023).

The prominence of “General IPV Resources and Support” websites can be attributed to its ability to provide immediate, actionable connections to established support systems, including hotlines, shelter directories, legal advocacy services, and safety planning tools, creating safety nets beyond the forum itself. These resources are general and comprehensive, making them broadly applicable to diverse IPV help-seeking scenarios, and their affiliation with well-established institutions enhance their perceived credibility. Moreover, these resources typically require less specialized knowledge from online health community members to recommend and are more commonly shared. Less commonly shared are targeted resources like specialized legal advice websites and specific safety planning strategies which may require legal interpretation or personalized risk assessments. The high prevalence of “Understanding IPV” websites reflect the fundamental need for survivors to contextualize their experiences within broader patterns and social dynamics of abuse. Many survivors struggle to recognize their experiences as abuse, influenced by perpetrators’ tactics and cultural misconceptions about IPV (Klencakova et al., 2023; Adams et al., 2013; Lederman et al., 2014). Prior research also found the societal normalization of dating abuse exacerbates IPV survivors’ self-blame and shame, severely impairing their ability to disclose experiences and seek external support (Kirkman et al., 2025). By sharing educational resources about power dynamics, warning signs, and abusive partners’ motives, online health community members help survivors validate their experiences and identify manipulation patterns they might overlook. These resources are particularly valuable in the early stages of help-seeking, when survivors are still assessing whether their relationships are abusive. Furthermore, many websites’ focus on the vicious cyclic nature of abuse reflects the reality that many survivors return to their abusive relationships multiple times. As such, education about these recurring cycles is critical throughout all stages of the leaving process.

Notably, the most popular resource within the ‘Understanding IPV’ category is the book *Why Does He Do That: Inside the Minds of Angry and Controlling Men* by Lundy Bancroft. Unlike many traditional methods of grasping knowledge, this book breaks down theoretical information about IPV into digestible anecdote-based sections (Bancroft, 2003). This preference suggests online health community users’ interest in explanatory content about abuse dynamics presented through relatable anecdotes. This finding reinforces previous studies that indicated that IPV survivors’ informational needs extended from practical education to theoretical understanding of abuse cycle and unhealthy power dynamics (Flynn and Graham, 2010; Wood, 2017). Considering that the general population may find psychiatric or academic terminology of textbooks and formal programs obscure, and some survivors have even suffered cognitive impairments caused by Post-Traumatic Stress Disorder (PTSD) and traumatic brain injuries (Klencakova et al., 2023; Adams et al., 2013), an anecdotal format may be more accessible to them. However, although online health community members are generally not clinically trained and often provide advice based on personal experiences, they may use these websites to gain and share theoretical knowledge that appears to be expert-validated (Lederman et al., 2014; Raphael, 2000). For example, users may “diagnose” their own partners or other users’ partners as having ‘narcissistic personality disorder’ based on popular psychology articles, or oversimplify complex concepts like ‘trauma bonding’ into simplistic explanations that lack the nuanced understanding required for proper application. This raises concerns about potential transmission of pseudoscientific information. Interestingly, while websites like https://www.thehotline.org also provide educational content about abuse dynamics, peer supporters more frequently shared Bancroft’s book when addressing survivors’ need to understand IPV. This preference may reflect the perceived value of narrative-based content over institutional resources in peer support contexts, where personal relatability often takes precedence over clinical authority.

Furthermore, a concerning finding is the significant underrepresentation of safety planning resources despite their critical importance in IPV intervention. “Safety Warnings” and “Escaping an IPV Situation” combined constituted only 11.8% of shared resources, though safety planning is recognized as a cornerstone in IPV research and support (McFarlane et al., 2004; Sabri et al., 2018; Campbell, 2001; Wood et al., 2021). Safety planning—the process by which survivors assess risks, evaluate needed supports, and develop strategies to prevent future harm (Sabri et al., 2022)—plays a crucial role in survivor protection. Nevertheless, its limited visibility in online health community resource sharing suggests a significant gap in informal support networks.

This gap may stem from multiple factors. First, original posters often seek validation of their experiences by questioning whether their situation constitutes abuse, rather than requesting proactive safety planning strategies (Hui et al., 2023). Second, experienced survivors may have already implemented basic safety plans and instead, seek help with other specific challenges like housing, legal protections or child custody (Hui et al., 2023). Third and more critically, online health community members may lack familiarity with comprehensive safety planning and hence, default to simplified recommendations, such as encouraging original posters to leave their relationship without assessing their danger level or offering guidance on how to protect their safety. This tendency is particularly alarming given research establishing that leaving an abusive relationship represents the period of highest homicide risk for survivors (Klaus and Rand, 1984; Bernard and Bernard, 1983; Hulley et al., 2023). Online health community users may underestimate the substantial dangers and challenges associated with leaving an abusive relationship, allowing them to suggest exit strategies without adequate safety precautions. Meanwhile, they may also overlook the value of sharing harm reduction approaches (i.e., strategies that aim to minimize immediate risks and increase survivor safety) with survivors who choose to remain with abusive partners. This pattern suggests a critical need for IPV organizations and clinicians to expand safety planning education beyond formal service settings into informal survivor networks where dangerously simplified “just leave” advice may predominate without recognition of associated lethal risks.

### 4.3 Domains of shared website resources

While examining the website categories provides insight on the content shared, our analysis of website domains offers perspective on where this information originates. The most common domains of shared websites were .org (54.7%) and .com (27.6%). These sites frequently shared information belonging to the category of “General IPV Resources and Support” for IPV survivors. Most .org websites correspond to IPV protection agencies and non-profit organizations (NGOs), providing comprehensive information on identifying abuse and accessing crucial services like legal help, shelter and counseling (DVSAS, n.d.), while the .com websites generally contained news, blogs, and informational articles about IPV (DVSAS, n.d.; Strangulation Training Institute, n.d.; Reddit, n.d.b; WBTV, n.d.). The .org and .com domains may have had the largest percentage of shared website URLs because they contained the widest variety of IPV websites, by content, type, and source, including resources from NGOs, websites that share other survivors’ IPV experiences, and blog posts. The breadth of support offered by diverse resources within these two domains could likely meet the needs of most IPV survivors (Cosijn and Ingwersen, 2000). The prevalence of organizational homepages (31.4%) among shared resources indicates online health community members frequently direct help-seekers to comprehensive resource hubs rather than specific information pages. This approach aligns with research on coordinated community responses (CCRs) for IPV survivors, which integrate multiple services from education to healthcare and legal support (Shorey et al., 2014; Greeson and Campbell, 2013). Such comprehensive resources may benefit survivors by reducing the burden of locating each service separately, suggesting that specialized programs should consider developing more integrated resource models.

Despite this domain distribution pattern, no significant association was found between domain type and resource relevance to original poster’s needs, which means .org websites from nonprofit organizations were not statistically more likely to address users expressed needs than websites from .com or other domains. This finding may be explained by several factors. First, resource relevance depends more on content alignment with survivors’ specific needs than institutional affiliation. Content providing clear, actionable guidance may be equally valuable regardless of whether it originates from NGOs or commercial platforms. Second, traditional boundaries in IPV resource development have shifted, as commercial media increasingly engage IPV experts to develop content, while some NGO websites offer generalized rather than personalized information. Third, online health community members’ link-sharing decisions may be influenced by factors including search result visibility, URL accessibility, and personal usage experience, rather than domain considerations alone. These findings suggest that when evaluating IPV online resources, professionals should prioritize content quality indicators such as accuracy, timeliness, and relevance over domain-based quality assumptions.

### 4.4 Relevance of shared resources

The finding that 75.3% of shared resources addressed original posters’ expressed needs demonstrates the general effectiveness of peer support in online health communities. However, we found a lower relevance rate (57%) for resources related to financial support, which aligns with growing concerns about economic, or financial, IPV identified in recent research. Economic abuse, as a distinct form of IPV, impedes victims’ ability to achieve financial independence and escape abusive partners, yet it is often overlooked in online discussions and resources. Similarly, the relatively low relevance rate for sexual abuse-related resources (61.5%) suggests potential resource gaps or sharing barriers in these sensitive domains. Several factors may contribute to these lower relevance rates. First, specialized online resources addressing economic and sexual abuse remain relatively scarce compared to those for physical abuse, limiting the options available for peer supporters to share. Previous studies also mentioned existing platforms inadequately address economic and sexual abuse (Postmus et al., 2018; Campo and Steinert, 2020). In addition, stigma and discomfort surrounding sexual abuse may act as a substantial barrier that inhibits open dialogue. Sowersby et al. (2022) conducted a discourse analysis of Twitter data regarding “rough sex” defense cases, finding that online discussions featured victim-blaming, normalized male sexual entitlement, and portrayed victims as complicit. Similarly, Williams et al. (2023) analyzed Twitter users’ reactions to the retrial of a footballer accused of rape and found that fan loyalty often prioritized the accused athlete’s career over the victim’s experience, amplifying hostility toward survivors. This discursive environment may discourage survivors from disclosing their experiences or seeking support. Research indicates that individuals often hesitate to discuss sensitive issues due to fear of judgment or misunderstanding from peers (Ryu and Pratt, 2025). Consequently, peer supporters may preferentially share general resources rather than addressing the issues directly. These findings highlight the need to expand resource coverage to ensure comprehensive responses to all manifestations of IPV, particularly those that may not be as readily identifiable as physical violence but are equally destructive. Developing accessible, shareable resources specifically addressing economic and sexual abuse could better equip peer support networks to meet these needs.

Additionally, there was no association found between the characteristics of the posts (e.g., post length, specificity of questions), the characteristics of the shared resources (e.g., website domain, category of website content), and the relevance of websites to original posters’ needs. This could be partially explained by the large proportion of web resources falling within the category of “General IPV Resources and Support.” Because these general IPV sites provide a broad range of IPV information, strategies, and resources, they can be counted as relevant for most original poster requests, while they were not always coded as relevant. For example, if an original poster requested a specific category of help (e.g., housing support), and an online health community user shared the home page of a website that contains general information about many domains, including housing support, then the website would have been annotated as “General IPV Resources and Support,” while original poster’s need would have been categorized under “Financial, Healthcare, Housing, and Food Support.” Hence, since the type of help sought would be categorized differently from the type of help provided, our results would incorrectly indicate the website as being “not relevant” to original poster’s needs. Therefore, using a multilabel system wherein posts and websites can be coded as multiple domains would be useful for a future iteration of this study.

### 4.5 Clinical implications

The findings from this study have several implications for healthcare providers and IPV service professionals. The high relevance rate (75.3%) of resources shared in IPV online health communities validates these platforms as valuable information sources for survivors, particularly those facing barriers to formal support services. Clinicians should recognize that many survivors access online peer support independently, particularly those reluctant to disclose in clinical settings. It is important to develop high-quality online resources that survivors can either find on their own or be discreetly signposted toward. Healthcare providers can utilize our detailed categorization of website content to better understand the types of information IPV survivors seek and receive online. This knowledge can inform development of resources addressing domains where online support appears inadequate, particularly finance, housing, and healthcare.

The limited representation of safety planning resources highlights a critical area for clinical attention. Healthcare providers should recognize that even when survivors disclose high-risk factors like strangulation, they may not automatically receive appropriate safety planning resources in online communities. This underscores the essential role clinicians play in conducting formal danger assessments and developing comprehensive safety plans, particularly for survivors in high-risk situations. However, given the scale and real-time nature of online health communities, manual identification of all high-risk cases by clinicians may not be feasible. Emerging artificial intelligence technologies may help address these challenges. A recent study by Guan et al. (2025) developed a fine-tuned large language model and found it can automatically classify IPV information needs with 70.49% accuracy while significantly reducing processing time, potentially enabling more timely automatic identification of survivors requiring urgent safety planning resources. Moreover, our findings regarding the lower relevance rates for sexual abuse and economic abuse resources suggest that medical professionals should pay special attention to these frequently overlooked forms of IPV and develop targeted resources to fill this gap.

For IPV service organizations, our findings suggest opportunities to enhance the online accessibility of safety planning resources. The underrepresentation of these critical resources in online health communities indicates a need for broader dissemination of harm reduction strategies beyond professional spaces to include informal survivor networks. Additionally, the finding that “General IPV Resources and Support” achieved higher relevance rates than specialized service categories supports the development of integrated resource models that reduce the burden on survivors to locate individual services separately.

### 4.6 Limitations

The results of this study should be interpreted and applied carefully. First, Reddit predominantly attracts English-speaking users, restricting our dataset’s representation of cultural backgrounds to predominantly Western perspectives and experiences. Cultural factors significantly influence IPV stigmatization, help-seeking behaviors, and resource availability, limiting the applicability of our findings across diverse populations. Second, our sample was restricted to self-identified women over 18 years of age who actively sought help through this specific subreddit, so the findings cannot be generalized to male survivors or those who sought help through other abuse-focused communities. Moreover, this study depended on the details provided in users’ initial posts during the screening procedure to determine whether the original poster was female and aged >18 years. However, some users may have hidden their actual sex and age because of privacy concerns. Furthermore, the anonymity features of Reddit also prevented extraction of demographic information, precluding analysis of how demographic characteristics might influence help-seeking patterns or resource relevance. Additionally, while Reddit attracts users globally, our analysis revealed that shared resources were predominantly US-specific rather than universally applicable. Most practical resources such as hotlines, shelter directories, and legal aid were tailored to the US context, potentially limiting their generalizability for survivors in other countries with different IPV support systems and legal frameworks. Furthermore, multiple statistical comparisons were conducted without correction for multiple testing, and our study had limited power to detect small effect sizes. Future studies with larger sample sizes are warranted to examine whether the descriptive differences observed in this study represent true associations between post characteristics and the relevance of shared resources.

## 5 Conclusion

This study provides valuable insights into the landscape of website resources shared within IPV online health communities, examining their categories, domains, and relevance to help-seekers’ needs. Our findings demonstrate that 75.3% of shared resources address the expressed needs of help-seeking survivors, validating these digital platforms as potentially valuable information sources. Online health community members focus on providing general resources and educational content for foundational understanding, but gaps in practical support categories suggest areas for improvement. The absence of clear associations between post characteristics, domain types, and resource relevance challenges simplistic approaches to online resource evaluation. These findings have important implications for healthcare providers, IPV service organizations, and online community moderators, suggesting opportunities to enhance digital support systems through targeted resource development in underserved domains, particularly safety planning and practical assistance areas. Future research examining resource accuracy, effectiveness, and user experiences would further strengthen our understanding of how digital platforms can optimally support IPV survivors throughout their help-seeking journeys.

## Data Availability

Due to the sensitive nature of intimate partner violence discussions and ethical considerations for participant privacy, the annotated research dataset is not publicly shared. Additional details regarding the annotation procedures are available from the corresponding author upon reasonable request.
